# High orange juice consumption with or in-between three meals a day differently affects energy balance in healthy subjects

**DOI:** 10.1038/s41387-018-0031-3

**Published:** 2018-04-25

**Authors:** Franziska A Hägele, Franziska Büsing, Alessa Nas, Julian Aschoff, Lena Gnädinger, Ralf Schweiggert, Reinhold Carle, Anja Bosy-Westphal

**Affiliations:** 1 Institute of Nutritional Medicine, University of Hohenheim, Stuttgart, Germany; 2 Institute of Human Nutrition and Food Sciences, Christian-Albrechts-University, Kiel, Germany; 3 Institute of Food Science and Biotechnology, University of Hohenheim, Stuttgart, Germany; 4 Biological Science Department, King Abdulaziz University, Jeddah, Saudi Arabia

## Abstract

Sugar-containing beverages like orange juice can be a risk factor for obesity and type 2 diabetes although the underlying mechanisms are less clear. We aimed to investigate if intake of orange juice with or in-between meals differently affects energy balance or metabolic risk. Twenty-six healthy adults (24.7 ± 3.2 y; BMI 23.2 ± 3.2 kg/m^2^) participated in a 4-week cross-over intervention and consumed orange juice (20% of energy requirement) either together with 3 meals/d (WM) or in-between 3 meals/d (BM) at ad libitum energy intake. Basal and postprandial insulin sensitivity (primary outcome), daylong glycaemia, glucose variability and insulin secretion were assessed. Body fat mass was measured by air-displacement plethysmography. After BM-intervention, fat mass increased (+1.0 ± 1.8 kg; *p* < 0.05) and postprandial insulin sensitivity tended to decrease (ΔMatsuda_ISI_: −0.89 ± 2.3; *p* = 0.06). By contrast, after WM-intervention fat mass and gamma-glutamyl transferase (GGT) decreased (−0.30 ± 0.65 kg; −2.50 ± 3.94; both *p* < 0.05), whereas glucose variability was higher (ΔMAGE: +0.45 ± 0.59, *p* < 0.05). Daylong glycaemia, insulin secretion, changes in basal insulin sensitivity, and triglycerides did not differ between WM- and BM-interventions (all *p* > 0.05). In young healthy adults, a conventional 3-meal structure with orange juice consumed together with meals had a favorable impact on energy balance, whereas juice consumption in-between meals may contribute to a gain in body fat and adverse metabolic effects.

## Introduction

A high intake of sugar-containing beverages is associated with overweight and obesity^1^ and is therefore suspected to promote weight gain. Increased consumption of fruit juice was indeed associated with 4-year weight gain similar to increased intake of fruit punch^2^. Since 100% fruit juice has a comparable sugar-content to sugar-sweetened beverages (SSB), the consumption of fruit juice may also be associated with an increased risk for type 2 diabetes^3^. Fruit juices contain important nutrients (i.e., vitamin C, potassium, folate, magnesium, and ß-carotene^4^) and flavonoids, and are an important contributor to total fruit intake^5^. Whereas orange juice has long been an integral part of a traditional (continental and American) breakfast, soft drinks are usually consumed to satisfy one’s thirst. Consequently, the effect of orange juice consumption with meals versus in-between meal ingestion should be investigated regarding the effect of energy balance and metabolic risk.

Several mechanisms have been proposed to explain the association between consumption of sugar-containing beverages and weight gain. First, it has been shown that the consumption of energy-dense beverages does not lead to a lower food intake in an acute meal setting, thus leading to a higher total energy intake^6–9^. This is also true for observational studies where the energy consumed from caloric beverages was not compensated by reducing solid food intake^10, 11^. These findings were explained by a low satiety value of beverages (for review see^12^). Fruit juice contains very little or no fiber, and has been shown to be less satiating than whole fruits^13^. In line with this finding, in contrast to whole fruits, consumption of fruit juices has been shown to promote long term weight gain^14^. The second proposed mechanism is known as the “carbohydrate-insulin theory of obesity” and is based on the high glycemic index of sugar-containing drinks, leading to postprandial hyperinsulinemia and thus promoting fat storage and inhibiting fat oxidation^15, 16^. The rapid decline in blood glucose levels caused by postprandial hyperinsulinemia is also believed to increase appetite and thus subsequent energy intake^16^. Both effects, remain however insufficiently substantiated by scientific evidence and thus are highly controversial (for review see^17, 18^).

Intake of sugar-containing drinks with or in-between meals may differently affect metabolic regulation, because postprandial glycemia is lowered especially by protein content of the diet, which is due to slower gastric emptying and enhanced insulin response^19^. On the other hand, intake of juice in-between main meals (e.g., snacking behavior) may prevent the drop in insulin between meals and thus may inhibit effective lipid oxidation. According to the “carbohydrate-insulin theory of obesity” fasting periods in-between meals are important, because the decrease in insulin levels activates lipolysis and lipid oxidation which may improve fat balance^15^. In addition, increased meal frequency is suggested to be a risk factor for increased energy intake^20^. We therefore hypothesized that in-between meal consumption of fruit juice leads to a positive energy balance and increases metabolic risk, whereas intake of juice together with three main meals can prevent these adverse effects.

## Methods

### Study population

Twenty-six healthy adults (13 women, 13 men) aged between 20 and 45 years were recruited in April and September 2016 by notice board postings at the Universities of Hohenheim and Stuttgart. Exclusion criteria were daily consumption of fruit juice or SSB, fructose intolerance, habitual meal skipping, chronic diseases, regular use of medication or supplements, alternative eating habits and smoking. The study protocol was approved by the ethics committee of the State Medical Council of Baden-Württemberg, Germany. This trial was registered at clinicaltrials.gov under NCT02974478. All subjects provided written informed consent before participation. In a previous study we observed a 17% difference in postprandial insulin sensitivity after consumption of high vs. low GI-SSBs^21^. Twenty-five participants are therefore needed to detect a difference in Matsuda-index of similar magnitude between orange juice consumption with and in-between meals (assuming a power of 80% and an alpha level of 5%).

### Study protocol

**Fig. 1:**
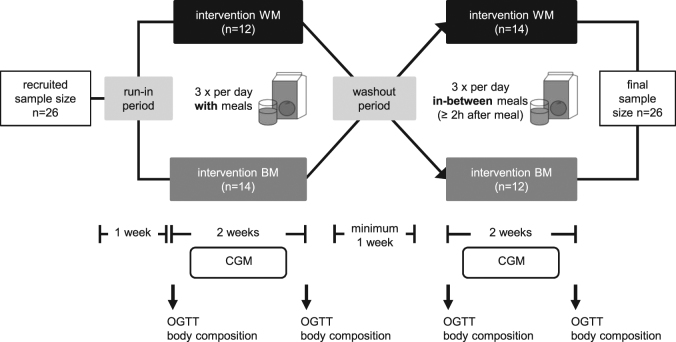
Flow diagram of the cross-over study protocol. *BM* orange juice in-between meals, *CGM* continuous glucose monitoring, *OGTT* oral glucose tolerance test, *WM* orange juice with meals

A cross-over free-living nutrition intervention was conducted at the Institute of Nutritional Medicine, University of Hohenheim in Stuttgart, Germany. An outline of the study protocol is given in Fig. 1. The primary outcome was insulin sensitivity and the secondary outcome body fat mass. Prior to the intervention, subjects kept a nutrition diary to assess normal nutrition habits and were asked to maintain their habitual choice of foods throughout the study. During a 1 week run-in period and the subsequent entire intervention, subjects were instructed to avoid the consumption of citrus fruits, additional orange juice and SSB in order to obtain equal baseline conditions and avoid confounders. A minimum of 1 week washout period was used between the 2-week consumption of each intervention period. Participants in the first group recruited in April began with the with meal (WM)-intervention, while participants in the second group recruited in September began with the in-between meal (BM)-intervention. Throughout intervention periods, participants were asked to consume only three meals a day and drink orange juice three times a day either with meals (WM) or in-between meals (BM) for two weeks. During the BM-intervention, participants were instructed to drink the orange juice not less than 2 h after meals. Participants came to the institute for visits at the beginning (day 1) and end (day 15) of each intervention phase. On these four visiting days (T1–T4) body composition was measured and an oral glucose tolerance test was performed. Subjects were asked to go to bed before midnight and to abstain from vigorous activity and alcohol consumption prior to visiting days to avoid potential bias on glucose tolerance.

### Intervention with orange juice

The orange juice was 100% juice with pulp, containing 43 kcal/100 mL and 8.9 g sugar (3.2 g sucrose, 2.7 g glucose and 3.0 g fructose)/100 mL according to the manufacturer and as verified by own analyses. The amount of orange juice provided was defined as 20% of individual energy requirement. Energy requirement was assessed by multiplying resting energy expenditure (REE, predicted according to^22^) by physical activity level (PAL). Subjects were interviewed about their habitual daily activity and an individual PAL between 1.4 (low active) and 1.8 (active) was estimated^23^. The individual amount of orange juice was calculated at the beginning of each intervention phase to meet 20% of daily energy requirement. The amount of orange juice per serving was weighted in bottles and the filling line was marked. For each serving, subjects refilled the bottles up to the marking. All orange juice was provided by the Institute of Nutritional Medicine.

### Fasting and postprandial glucose metabolism

On visiting days, subjects came to the Institute between 6:30 a.m. and 8:00 a.m. Blood samples were collected after an overnight fast (≥10 h) and 30, 60, and 120 min after a standard oral glucose tolerance test (OGTT, intake of 75 g glucose, Accu-Chek® Dextrose O.G-T., Roche Diagnostics GmbH, Mannheim, Germany). Basal insulin sensitivity was calculated using Homeostatic Model Assessment-insulin resistance (HOMA-IR = fasting glucose (mg/dl) x fasting insulin (μU/mL) /405;^24^), which is a valid index for the assessment of hepatic insulin sensitivity^25^. Postprandial insulin sensitivity was assessed by Matsuda Insulin Sensitivity Index (Matsuda_ISI_ = 10,000/√((fasting glucose x fasting insulin) x (mean glucose x mean insulin during OGTT))), which is a validated index and was developed especially for the assessment of insulin sensitivity using OGTT-data^26^. Interstitial glucose concentrations were measured by continuous glucose monitoring (CGM, Dexcom G4 Platinum, Nintamed GmbH & Co KG, Mainz, Germany) for 7 days during each intervention. The sensor was placed at the back of the upper arm to monitor glucose levels in the subcutaneous tissue. Sensor readings were reported every 5 min. CGM-devices were calibrated twice a day against fasting capillary blood samples. Area under the curve (AUC) was calculated as incremental AUC (iAUC) for 18 h (6:00–00:00 a.m.) from 3–5 valid daylong CGM-data sets using trapezoidal rule^27^. Daylong glucose variability was determined by mean amplitude of glycemic excursions (MAGE) using 24-h CGM-data:

$${\sum} {\frac{\lambda }{\chi }\ {\mathrm{with}}\ \lambda > \gamma }$$

where *λ* is the difference from peak to nadir, *χ* is the number of valid observations, and *γ* is 1 SD of mean interstitial glucose values in a 24-h period^28, 29^ using a published macro^30^. Fasting fructosamine was determined to asses average glycemia during the preceding 1–3 weeks^31^. Daylong insulin secretion was assessed by 24-h urinary C-peptide excretion at the end of each intervention phase.

### Blood sampling and analytical methods

Blood sampling was conducted by vein cannula. Plasma glucose was determined using hexokinase method, and serum insulin and urinary C-peptide excretion were measured by electrochemiluminescence immunoassay. Serum gamma-glutamyl transferase (GGT), triglycerides (TG) and serum fructosamine were measured by photometry before and after each intervention phase, respectively.

### Body composition

Height was measured using a stadiometer (seca 274, seca GmbH & Co.KG, Hamburg, Germany). Body composition was assessed from the mean of duplicate measurements using air displacement plethysmography (ADP) by the BodPod^TM^ Body Composition System (COSMED USA, Inc., Concord, CA, USA) at the beginning and end of each intervention phase. ADP is based on the assessment of body density from weight (Tanita scale coupled to the BodPod^TM^-System) and body volume and the subsequent calculation of percentage fat mass (FM) based on assumed constant densities of fat-free mass and FM. Minimal detectable change (MDC) of FM measured by ADP (BodPod^TM^ device) was 0.53 kg as assessed from duplicate measurements in 7 subjects (BMI: 19.6–25.0 kg/m^2^; FMI: 3.9–7.2 kg/m^2^). Fat mass index (FMI) was calculated as FM divided by height squared (kg/m^2^)^32^. Weight was assessed by an electronic scale coupled to the BodPod^TM^ system.

### Physical activity

Physical activity was continuously measured using a triaxial activity monitor (ActivPAL^TM^, Paltechnologies Ltd., Glasgow, UK). The ActivPAL^TM^ was fixed on the upper thigh with waterproof patches and was worn permanently during both intervention periods. Subjects were requested to maintain physical activity constant for the whole study period and to refrain from exercise one day prior visits.

### Statistical analyses

Data analyses were performed using SPSS version 22.0 (SPSS Inc., Chicago, IL, USA). Data are presented as mean ± SD. The assumption of normality was verified with the Kolmogorov–Smirnov test. Overall parameters HOMA-IR, GGT and TG as well as ΔFM and ΔHOMA-IR for BM-intervention did not meet the criteria of normal distribution. Differences between pre and post intervention (T1 vs. T2 and T3 vs. T4) as well as between changes with both interventions (ΔT2-T1 vs. ΔT4-T3) were analyzed by two-sided paired *t*-test or Wilcoxon-test, as appropriate. Differences in HOMA-IR and Matsuda_ISI_ between participants with a stable FM or a gain in FM, as well as differences between men and women were analyzed by Mann–Whitney-*U*-Test and independent *t*-test, respectively.

Relationships between normally distributed parameters were determined using Pearson correlation coefficients. Non-parametric relationships were analyzed using Spearman correlation coefficients. Power analysis was performed using the free-software G*Power 3.1.7. A *p*-value < 0.05 was considered to be statistically significant.

## Results

Thirteen women and thirteen men aged 20–33 years (24.7 ± 3.2 years) and with a BMI between 19.1–33.3 kg/m^2^ (23.2 ± 3.2 kg/m^2^) participated in this trial. According to WHO criteria five subjects were overweight and one participant was obese. The REE and therefore the intake of orange juice did not differ between both interventions (REE WM: 1662 ± 270 kcal/d vs. BM: 1663 ± 275 kcal/d; orange juice WM: 1277 ± 221 mL/d vs. BM: 1278 ± 224 mL/d; all *p* > 0.05). The amount of sugars provided by the orange juice was 112.3 ± 19.4 g/d in WM-intervention and 112.5 ±19.7 g/d in BM-intervention (difference not significant). During the entire BM-intervention, participants consumed 7697 ± 1349 kcal from orange juice which is equivalent to a theoretical gain in FM of 855 ± 150 g assuming an energy content of 9000 kcal/kg FM. Physical activity did not differ between both interventions (10,118 ± 3128 steps/d in WM vs. 10,528 ± 3438 steps/d in BM).

**Fig. 2:**
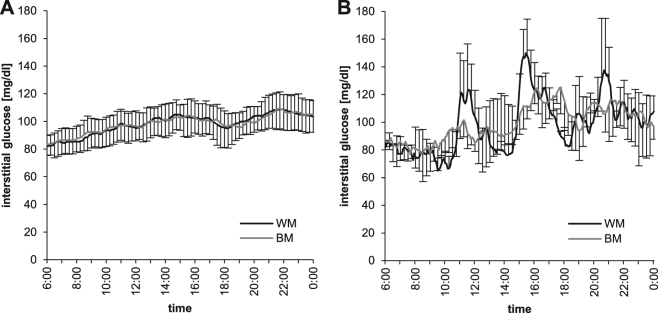
Daylong glycemia (CGM) for all participants (**A**, *n* = 26) and for one exemplary participant (**B**, *n* = 1) during interventions with orange juice with meals (WM) and in-between meals (BM). Glucose variability during the day is only visible in the individual day profile since exact meal times were not prescribed by the study protocol and glucose variability is therefore averaged out by the mean value of all subjects

As shown in Table 1, FM significantly decreased after the intake of orange juice WM and increased after the BM-intervention (both, *p* < 0.05), whereas body weight did not change. During BM-intervention, 11 participants (5 women and 6 men) had an FM gain greater than the MDC (0.53 kg), whereby women gained more FM compared to men (+3.92 ± 2.06 vs. +1.30 ± 0.59 kg FM; *p* < 0.05). GGT also decreased with WM, whereas no changes were observed with BM-intervention. TG, postprandial and basal insulin sensitivity remained unchanged with both interventions. There was a tendency for a decrease in basal insulin sensitivity (*p* = 0.06) with ΔWM compared to ΔBM (Table 1). This is however likely explained by higher baseline levels in HOMA-index at WM-intervention compared to BM-intervention (*p* < 0.05), because higher baseline levels were associated with a larger decrease in HOMA-index due to WM-intervention (*r* = −0.62, *p* < 0.01). No differences in changes of Matsuda_ISI_ and HOMA-IR were observed between subjects with stable FM and a gain in FM after BM-intervention. During WM-intervention, there was a tendency for a positive correlation between ΔFM and ΔMatsuda_ISI_ (*r* = 0.38; *p* = 0.06), whereas this correlation was not observed with BM-intervention. MAGE-index and the maximum glucose value were significantly higher during WM-intervention when compared with BM-intervention, whereas iAUC glucose, C-peptide excretion (Table 2) and fructosamine (Table 1) did not differ between both interventions. Time course of daylong glycemia is shown in Fig. 2 for all participants (panel A) and illustrated for one exemplary participant (panel B) to demonstrate the higher glucose variability during WM-intervention as compared to BM-intervention. This variability is averaged out by the mean value from all subjects, because exact meal times were not prescribed by the study protocol. Although the BMI range in our population was 19.1–33.3 kg/m^2^, there were no correlations between BMI and the changes in FM, Matsuda- and HOMA-index, GGT, iAUC glucose, MAGE-index and C-peptide excretion.

**Table 1:** Comparison of energy balance and metabolic risk between interventions with orange juice with meals and in-between meals^a^

	WM			BM			∆BM-WM
	T1	T2	∆T2-T1	T3	T4	∆T4-T3	∆∆(T4-T3)–(T1-T2)
Energy balance							
weight, kg	70.3 ± 14.1	70.4 ± 14.4	+0.13 ± 0.82	70.1 ± 14.5	70.3 ± 14.5	+0.21 ± 0.94	NS
FM, kg	15.3 ± 6.8	15.0 ± 6.7	−0.30 ± 0.65*	14.1 ± 6.3	15.1 ± 6.6	+1.02 ± 1.81**	*p* = 0.001
FMI, kg/m^2^	5.15 ± 2.3	5.05 ± 2.2	−0.10 ± 0.22*	4.78 ± 2.1	5.08 ± 2.2	+0.30 ± 0.58*	*p* = 0.019
Metabolic risk							
HOMA-IR	1.97 ± 1.1	1.79 ± 1.0	−0.18 ± 0.65	1.62 ± 0.8	1.63 ± 0.6	+0.01 ± 0.67	*p* = 0.062
Matsuda_ISI_	6.01 ± 2.7	6.08 ± 2.7	+0.07 ± 1.92	7.11 ± 2.9	6.21 ± 2.3	−0.89 ± 2.33	NS
Fructosamine, µmol/L	236.1 ± 25.3	234.0 ± 15.9	−2.0 ± 20.2	233.3 ± 13.0	236.0 ± 15.6	2.7 ± 15.7	NS
GGT, U/L	17.9 ± 7.9	15.4 ± 6.7	−2.50 ± 3.94**	16.9 ± 7.3	16.4 ± 6.6	−0.42 ± 4.49	NS
TG, mg/dL	100.9 ± 57.0	98.5 ± 47.7	−2.35 ± 45.83	87.9 ± 42.0	89.8 ± 34.8	+1.92 ± 33.80	NS

*BM* orange juice in-between meals, *FM* fat mass, *FMI* fat mass index, *GGT* gamma-glutamyl transferase, *T1* study day before WM-intervention, *T2* study day after WM-intervention, *T3* study day before BM-intervention, *T4* study day after BM-intervention, *WM* orange juice with meals

^a^Values are means ± SDs, *n* = 26

*p < 0.05; **p < 0.01, *p*aired *t*-test or Wilcoxon-test

**Table 2:** Comparison of daylong glycemia and insulin secretion between interventions with orange juice with meals (WM) and in-between meals (BM)^a^

	WM	BM	∆BM-WM
iAUC glucose, mg/dL x 18 h	289 ± 89	260 ± 72	−29.56 ± 93.55
MAGE	2.08 ± 0.3	2.52 ± 0.5	+0.45 ± 0.59**
MAX glucose, mg/dL	151 ± 12	143 ± 13	−7.88 ± 16.37*
C-peptide, µg/d	70.0 ± 37.0	70.6 ± 41.9	+0.52 ± 39.19

*iAUC* incremental area under the glucose curve for 18 h, *MAGE* mean amplitude of glycemic excursions, *MAX glucose* maximal interstitial glucose value per day

^a^Values are means ± SD, *n* = 26

*p < 0.05, **p < 0.01, *p*aired *t*-test

## Discussion

In line with our hypothesis, the present study demonstrates that the impact of orange juice consumption on energy balance and metabolic risk depends on timing of juice intake in relation to meals. When compared with in-between meal consumption, intake of orange juice together with breakfast, lunch and dinner prevented a positive energy balance and even lead to a loss of body fat mass when no snacks were consumed in-between meals (Table 1). The effect of orange juice consumption on risk of overweight and obesity is controversial with some studies showing a higher risk^2, 33–35^, whereas others reported no effect^36–38^ or even found a lower body weight in regular fruit juice consumers compared to people with no fruit juice intake^39, 40^. Associations between beverage intake and weight status observed in epidemiological studies are, however, not necessarily causal. Lifestyle behavior may differ between different types of beverage consumers and therefore act as an important confounder. Accordingly, 100% orange juice consumption was found to be associated with better diet quality, improved nutrient adequacy, and improved biomarkers of health in adults^41^. In contrast, intake of SSB was associated with a less healthy diet^42^ and high soft drink consumers also engaged in less physical activity than low soft drink consumers^2, 43^.

Similar to the above mentioned controversial findings of cross-sectional and observational data, evidence from intervention studies on orange juice and weight gain is inconclusive. A previous study has shown that consumption of 250 mL orange juice per day for a period of 12 weeks did not adversely affect body weight or insulin sensitivity^44^. This was however also true for the control group who consumed an energy and sugars-matched orange-flavored drink. In another dietary intervention trial, body weight and leptin levels decreased in overweight and obese subjects after consumption of 2 × 250 mL orange juice per day for a period of 12 weeks^45^. However, participants received nutritional advice to aid them in balancing out the additional calories that they were ingesting from orange juice intake. Unfortunately, it was not reported whether the subjects drank the orange juice with or in-between meals. One important difference between SSB and fruit juices is that juice is usually not consumed as a thirst quencher. Timing of consumption, i.e., with or in-between meals may therefore also be different (e.g., drinking orange juice as part of a breakfast). Unlike numerous other studies, one observational study in Canadian children specifically asked for SSB consumption in-between meals and found that this intake pattern more than doubles the odds of being overweight^34^.

Contrary to previous interventions^44, 45^, we used a very high amount of orange juice (about 1.28 L/d) in order to achieve a measureable impact on energy balance within the short time frame. Compared with SSB, orange juice is usually consumed in smaller amounts of only 7.8 L per capita per year (equivalent to 21 mL per day^46^,) in Germany. This may partly explain the lack of association between orange juice consumption and body weight in some studies. We have chosen an amount of orange juice that closely resembles the habitual intake of soft drinks in young German adults. The highest amounts are consumed by young 18–29 year old men with about 900 mL of SSB and fruit juices per day^47^. This equals a habitual intake of sugar of about 96 g/d from SSB and fruit juice, being comparable to the consumed amount of sugar from orange juice in the present study. During BM-intervention, participants in the present study consumed a total of 7697 ± 1349 kcal from orange juice. Without compensation during the following meals, this would lead to a positive energy balance and equals a theoretical gain in FM of 855 ± 150 g. Noteworthy; participants of the present study were not instructed to adapt their meals to compensate for the additionally consumed orange juice. The actual gain in FM of 1.02 ± 1.81 kg during BM-intervention confirmed this lack of caloric compensation during subsequent meals when orange juice is consumed as a snack in-between meals.

In-between meals, insulin levels decrease, and activate lipolysis that may also be important for metabolic regulation^15^. This is supported by protocols comparing low and high meal frequencies at the same energy intake. A lower meal frequency was associated with lower liver fat during hypocaloric^48^ as well as hypercaloric diets^49^. In line with metabolic improvement and a suspected loss in liver fat, serum GGT decreased with loss in FM after WM-intervention (Table 1). GGT is associated with several cardiovascular risk factors and shows a dose-response relationship with incident diabetes, even within its normal range^50^.

Contrary to the finding of Meng et al. 2017, in the present study the incremental glucose area under the curve did not differ and MAGE-Index was higher with WM-intervention compared to BM-intervention. Hence we may deduce that postprandial glycemia is not attenuated by a concurrent meal when sugar is consumed in a liquid form that quickly passes the stomach into the intestine^19^. This is in line with no significant differences in fructosamine levels between the interventions (Table 1).

The impact of regular orange juice intake on the risk for type 2 diabetes is as controversial as its effect on the risk of obesity (for meta-analysis see^51^). Despite the high sugar content of orange juice, flavanones and vitamin C may even reduce cardiovascular^45^ and diabetes risk^52^, possibly due to their antioxidant and anti-inflammatory activities. These components might also compensate for increased oxidative stress associated with increased glucose variability^53^ that was shown in the present study by a higher MAGE index with WM-intervention (Table 2). Finally, the subjects of our study were young, healthy and mostly normal weight. The results can therefore not be transferred to overweight, obese, and elderly people or patients with type 2 diabetes, because impaired glucose metabolism might lead to a more pronounced impairment of postprandial insulin sensitivity with BM-intervention.

The crossover intervention is an advantage of the present study. An additional strength is the measurement of body composition with elaborate repeated measurement of ADP in order to increase MDC of fat mass. There are however some limitations that need to be addressed. Although all study participants followed an ad libitum diet, meal frequency was fixed. We therefore could not examine the voluntary intake of snacks in-between meals. Since orange juice adds to the calories consumed at meal-time^54^, the loss in FM after WM-intervention is not due to a lower energy intake with meals but likely explained by the fact that participants were not allowed to eat in-between main meals. Finally, the results of our study only refer to liquid calories and cannot be transferred to solid snacks eaten in-between meals. Although most of our participants were normal weight, our findings may be of special importance for prevention of weight gain in overweight people since the propensity of weight gain may be more pronounced in this group.

In conclusion, in order to take advantage of the health benefits of fruit juice, dietary counselling should advice to drink orange juice together with only 3 main meals rather than as an in-between meal snack. Further studies are required to verify these results and to investigate their transferability to timing of SSB intake. Moreover, studies in vulnerable groups like overweight and elderly individuals are needed to investigate the impact of SSB consumption with or in-between meals on outcome parameters like liver fat and insulin resistance.

## References

[1] Te Morenga L, Mallard S, Mann J (2012). Dietary sugars and body weight: systematic review and meta-analyses of randomised controlled trials and cohort studies. BMJ.

[2] Schulze MB (2004). Sugar-sweetened beverages, weight gain, and incidence of type 2 diabetes in young and middle-aged women. JAMA.

[3] Muraki I (2013). Fruit consumption and risk of type 2 diabetes: results from three prospective longitudinal cohort studies. BMJ.

[4] Aschoff JK (2015). Bioavailability of β-cryptoxanthin is greater from pasteurized orange juice than from fresh oranges—a randomized cross-over study. Mol. Nutr. Food Res..

[5] Rechkemmer G (2002). Fünf am Tag—Obst und Gemüse: die Gesundheitskampagne mit biss!. Onkologe.

[6] Almiron-Roig E, Drewnowski A (2003). Hunger, thirst, and energy intakes following consumption of caloric beverages. Physiol. Behav..

[7] DellaValle DM, Roe LS, Rolls BJ (2005). Does the consumption of caloric and non-caloric beverages with a meal affect energy intake?. Appetite.

[8] Harper A, James A, Flint A, Astrup A (2007). Increased satiety after intake of a chocolate milk drink compared with a carbonated beverage, but no difference in subsequent ad libitum lunch intake. Br. J. Nutr..

[9] Tsuchiya A, Almiron-Roig E, Lluch A, Guyonnet D, Drewnowski A (2006). Higher satiety ratings following yogurt consumption relative to fruit drink or dairy fruit drink. J. Am. Diet. Assoc..

[10] Flood JE, Roe LS, Rolls BJ (2006). The effect of increased beverage portion size on energy intake at a meal. J. Am. Diet. Assoc..

[11] Appelhans BM (2013). Beverages contribute extra calories to meals and daily energy intake in overweight and obese women. Physiol. Behav..

[12] Dennis EA, Flack KD, Davy BM (2009). Beverage consumption and adult weight management: A review. Eat. Behav..

[13] Flood-Obbagy JE, Rolls BJ (2009). The effect of fruit in different forms on energy intake and satiety at a meal. Appetite.

[14] Hebden L (2017). Fruit consumption and adiposity status in adults: a systematic review of current evidence. Crit. Rev. Food Sci. Nutr..

[15] Astrup A, Raben A (1995). Carbohydrate and obesity. Int J. Obes. Relat. Metab. Disord..

[16] Ludwig D (2002). The glycemic index: physiological mechanisms relating to obesity, diabetes, and cardiovascular disease. J. Am. Med. Assoc..

[17] Bosy-Westphal A, Hägele F, Nas A (2017). Impact of dietary glycemic challenge on fuel partitioning. Eur. J. Clin. Nutr..

[18] Niwano Y (2009). Is glycemic index of food a feasible predictor of appetite, hunger, and satiety?. J. Nutr. Sci. Vitaminol..

[19] Meng H, Matthan NR, Ausman LM, Lichtenstein AH (2017). Effect of macronutrients and fiber on postprandial glycemic responses and meal glycemic index and glycemic load value determinations. Am. J. Clin. Nutr..

[20] McCrory MA, Howarth NC, Roberts SB, Huang TTK (2011). Eating frequency and energy regulation in free-living adults consuming self-selected diets. J. Nutr..

[21] Kahlhöfer J, Karschin J, Silberhorn-Bühler H, Breusing N, Bosy-Westphal A (2016). Effect of low-glycemic-sugar-sweetened beverages on glucose metabolism and macronutrient oxidation in healthy men. Int J. Obes..

[22] Harris JA, Benedict FG (1918). A biometric study of human basal metabolism. Proc. Natl. Acad. Sci. USA.

[23] Brooks GA, Butte NF, Rand WM, Flatt JP, Caballero B (2004). Chronicle of the Institute of Medicine physical activity recommendation: how a physical activity recommendation came to be among dietary recommendations. Am. J. Clin. Nutr..

[24] Matthews DR (1985). Homeostasis model assessment: insulin resistance and beta-cell function from fasting plasma glucose and insulin concentrations in man. Diabetologia.

[25] Hattersley JG, Möhlig M, Roden M, Arafat AM, Loeffelholz CV, Nowotny P (2012). Quantifying the improvement of surrogate indices of hepatic insulin resistance using complex measurement techniques. PLoS ONE.

[26] Matsuda M, DeFronzo RA (1999). Insulin sensitivity indices obtained from oral glucose tolerance testing: comparison with the euglycemic insulin clamp. Diabetes Care.

[27] Matthews JN, Altman DG, Campbell MJ, Royston P (1990). Analysis of serial measurements in medical research. BMJ.

[28] Service FJ (1970). Mean amplitude of glycemic excursions, a measure of diabetic instability. Diabetes.

[29] Standl E, Schnell O, Ceriello A (2011). Postprandial hyperglycemia and glycemic variability: should we care?. Diabetes Care.

[30] Hill NR (2011). Normal reference range for mean tissue glucose and glycemic variability derived from continuous glucose monitoring for subjects without diabetes in different ethnic groups. Diabetes Technol. Ther..

[31] Malmström H, Walldius G, Grill V, Jungner I, Hammar N (2015). Fructosamine is a risk factor for myocardial infarction and all-cause mortality—longitudinal experience from the AMORIS cohort. Nutr. Metab. Cardiovasc Dis..

[32] VanItallie TB, Yang MU, Heymsfield SB, Funk RC, Boileau RA (1990). Height-normalized indices of the body’s fat-free mass and fat mass: potentially useful indicators of nutritional status. Am. J. Clin. Nutr..

[33] Dennison B, Rockwell H, Baker S (1997). Excess fruit consumption by preschool-aged children is associated with short stature and obesity. Pediatrics.

[34] Dubois L, Farmer A, Girard M, Peterson K (2007). Regular sugar-sweetened beverage consumption between meals increases risk of overweight among preschool-aged children. J. Am. Diet. Assoc..

[35] Shefferly A, Scharf RJ, DeBoer MD (2016). Longitudinal evaluation of 100% fruit juice consumption on BMI status in 2–5-year-old children. Pediatr. Obes..

[36] O’Neil CE, Nicklas TA (2008). A review of the relationship between 100% fruit juice consumption and weight in children and adolescents. Am. J. Lifestyle Med..

[37] Papandreou D, Andreou E, Heraclides A, Rousso I (2013). Is beverage intake related to overweight and obesity in school children?. Hippokratia.

[38] Beck AL, Tschann J, Butte NF, Penilla C, Greenspan LC (2014). Association of beverage consumption with obesity in Mexican American children. Public Health Nutr..

[39] Akhtar-Danesh N, Dehghan M (2010). Association between fruit juice consumption and self-reported body mass index among adult Canadians. J. Hum. Nutr. Diet..

[40] Pereira MA, Fulgoni VL (2010). Consumption of 100% fruit juice and risk of obesity and metabolic syndrome: findings from the National Health and Nutrition Examination Survey 1999–2004. J. Am. Coll. Nutr..

[41] O’Neil CE, Nicklas TA, Rampersaud GC, Fulgoni Iii VL (2012). 100% Orange juice consumption is associated with better diet quality, improved nutrient adequacy, decreased risk for obesity, and improved biomarkers of health in adults: National Health and Nutrition Examination Survey, 2003-2006. Nutr. J..

[42] Duffey KJ, Popkin BM (2006). Adults with healthier dietary patterns have healthier beverage patterns. J. Nutr..

[43] Kvaavik E, Andersen LF, Klepp KI (2005). The stability of soft drinks intake from adolescence to adult age and the association between long-term consumption of soft drinks and lifestyle factors and body weight. Public Health Nutr..

[44] Simpson EJ, Mendis B, Macdonald IA (2016). Orange juice consumption and its effect on blood lipid profile and indices of the metabolic syndrome; a randomised, controlled trial in an at-risk population. Food Funct..

[45] Rangel-Huerta OD (2015). Normal or high polyphenol concentration in orange juice affects antioxidant activity, blood pressure, and body weight in obese or overweight adults. J. Nutr..

[46] Sennewald, K., Heitlinger, K. Geschäftsbericht 2014 (Verbandes der Deutschen Fruchtsaft-Industrie e.V., Germany, 2015).

[47] Rabenberg, M., Mensink, G. Limo, Saft & Co—Konsum zuckerhaltiger Getränke in Deutschland (Robert-Koch-Institut, Abteilung für Epidemiologie und Gesundheitsmonitoring, Germany, 2013).

[48] Kahleova H (2014). Eating two larger meals a day (breakfast and lunch) is more effective than six smaller meals in a reduced-energy regimen for patients with type 2 diabetes: a randomised crossover study. Diabetologia.

[49] Koopman KE (2014). Hypercaloric diets with increased meal frequency, but not meal size, increase intrahepatic triglycerides: a randomized controlled trial. Hepatology.

[50] Ahmed MH (2007). Biochemical markers: the road map for the diagnosis of nonalcoholic fatty liver disease. Am. J. Clin. Pathol..

[51] Imamura F (2015). Consumption of sugar sweetened beverages, artificially sweetened beverages, and fruit juice and incidence of type 2 diabetes: systematic review, meta-analysis, and estimation of population attributable fraction. BMJ.

[52] Sun Q (2015). Urinary excretion of select dietary polyphenol metabolites is associated with a lower risk of type 2 diabetes in proximate but not remote follow-up in a prospective investigation in 2 cohorts of US women. J. Nutr..

[53] Monnier L (2006). Activation of oxidative stress by acute glucose fluctuations compared with sustained chronic hyperglycemia in patients with type 2 diabetes. JAMA.

[54] Panahi S, El Khoury D, Luhovyy BL, Goff HD, Anderson GH (2013). Caloric beverages consumed freely at meal-time add calories to an ad libitum meal. Appetite.

